# Characterization of Inflammatory Biomarkers in Palatal Tissue of Patients with Bilateral Cleft Lip and Palate

**DOI:** 10.3390/life16060990

**Published:** 2026-06-12

**Authors:** Georgijs Kuļibaba, Māra Pilmane

**Affiliations:** Institute of Anatomy and Anthropology, Riga Stradins University, Dzirciema Street 16, LV-1007 Riga, Latvia; mara.pilmane@rsu.lv

**Keywords:** cleft palate, Granulysin, Resistin, FCGR1A, NF-kß p65, CD68

## Abstract

Orofacial clefts are among the most common congenital craniofacial anomalies in the world. Immunity factors modulate response, inflammation, and healing in clefted tissue. This study aims to evaluate the levels of the pro-inflammatory biomarkers Granulysin, Resistin, FCGR1A, NF-kßp65, and CD68 to describe and understand the morphopathological basis of inflammation. The comparison was done between patient and control samples across milk and mixed dentition age groups. In total, 14 patient samples were analyzed with a total of 10 control samples to form two distinct control groups with milk dentition age and mixed dentition age. Samples were analyzed using light microscopy, and a semi-quantitative method of evaluation and comparison was used to determine the number of immunohistochemically positive structures of patient and control samples. Statistics included Spearman’s correlation and Fisher’s exact test to compare groups and detect significant differences. NF-kßp65 in the milk dentition age group (*p* = 0.043 for NF-kßp65 in connective tissue, *p* = 0.017 for NF-kßp65 in salivary glands), and FCGR1A and CD68 in the mixed dentition age group showed statistically significant differences in the expression of palatal tissues compared to the controls (*p* = 0.016 for FCGR1A in connective tissue, *p* = 0.048 for CD68 in epithelium). Spearman’s rank correlation revealed eight very strong correlations among several factors and one strong correlation between factors. The presence of many very strong and strong Spearman’s correlations among inflammatory factors in cleft-affected individuals suggests heightened signaling in these pathways. Furthermore, the difference in the inflammatory factor expression at different dentition ages suggests variation in the inflammation character with age.

## 1. Introduction

Orofacial clefts (OFCs) are among the most common craniofacial anomalies [1,2,3]. There are several types of clefts that can develop. Clefts can be classified based on the anatomical location (cleft lip only, cleft palate only, cleft lip and palate), based on the side of the defect (unilateral affecting only one side or bilateral affecting both sides), and based on the length of the defect (complete and incomplete) [1,2]. Bilateral cleft lip and palate, which is analyzed in this study, is rare, as the most common type of cleft lip and palate is unilateral [4]. The etiology of OFCs is complex and remains partially unclear. There are many candidate genes and loci with variable functions that are associated with OFCs [5,6,7,8].

Children with OFCs commonly experience chronic states of inflammation in the orofacial zone, which significantly disrupt healing and tissue remodeling [9,10]. Raised pro-inflammatory cytokine levels as well as raised local defensive factors have already been described in some studies [10,11,12]. This raises the question of what morphological base causes the inflammation to persist, what peptides and factors become predominant in the palate and lip tissue, and what proteins are responsible for the inflammation in the clefted tissue. The objective of this study was to analyze in this research the expression levels of pro-inflammatory proteins: Granulysin, Resistin, FCGR1A, NF-kßp65, and CD68.

Granulysin (GNLY) protein is a member of the saposin-like protein family. Its structure suggests a potential mechanism of action by which GNLY functions as a lytic molecule [13,14,15,16,17]. Stenger et al. (1998) first described that GNLY on its own kills a wide spectrum of cell-free bacteria, including Mycobacteria tuberculosis, two fungi (*C. albicans* and *C. neoformans*), and a parasite (*L. major*) [18,19,20]. GNLY levels in systemic inflammatory disease have been proven to be increased [21,22,23]. However, data on GNLY expression levels in cleft-lip and -palate tissue are currently lacking.

Resistin was originally described as an adipocyte-secreted hormone that induced insulin resistance in rodents. Increasing evidence indicates its important regulatory roles in various biological processes, including several inflammatory diseases [24]. Resistin is a peptide hormone that belongs to the class of cysteine-rich secreted proteins involved in modulating inflammation and immunity [25,26].

In humans, during inflammation, Resistin is produced mainly from macrophages; especially strong expression has been detected during monocyte differentiation to macrophage [27,28]. Resistin promotes inflammation by upregulating the production and secretion of inflammatory cytokines, especially in some pro-inflammatory conditions, such as obesity, metabolic syndrome, and type 2 diabetes mellitus [29]. The blockage of Resistin expression by NF-kß inhibitors has been proven to reduce inflammation in some chronic conditions such as rheumatoid arthritis [30]. Until now, there is no data on the Resistin expression level in OFC tissue.

The FCGR1A gene in humans encodes CD64, which is the sole functional high-affinity IgG Fc receptor [31]. CD64 plays a crucial role in immune responses by mediating phagocytosis, degranulation, and cytokine production. FCGR1A was first sequenced by Hullet & Hoghart (1998), who discovered that FCGR1A is one of three members of the related gene family located on chromosome 1 and often expressed on the surface of monocytes, macrophages, and dendritic cells [32]. During infection, CD64 expression on the surface of neutrophils can increase rapidly under stimulation of bacterial lipopolysaccharide, IL-12, Iγ, and granulocyte colony-stimulating factor [33]. CD64 has been associated with such inflammatory conditions as sarcoidosis, retinopathy, and others [33]. In addition, some recent studies reported a strong correlation between IL-37 and CD64 [34]. However, CD64 has not been described in OFC pathologies.

The transcription factor NF-kß is a critical regulator of many cellular processes, including cell survival and inflammation [35]. The expression of the NF-kß p65 subunit can be induced by many different factors, including cytokines [36], hormones [37], chemical compounds [38], cellular stress [39], and others [40,41].

NF-kßp65 has been shown to play a role in the regulation of many cellular processes, including cell inflammation. Binding of the p65 + p50 heterodimers is believed to induce the expression of numerous pro-inflammatory genes [42,43,44]. NF-kßp65 has also been shown to induce the expression of several pro-inflammatory cytokines such as TNFα, IL-1β, IL-12, and IL-6 [45,46]. There are no studies evaluating NF-kß p65 expression level in OFCs.

CD68 is a member of the lysosome-associated membrane protein (LAMP) family that is restricted in its expression to cells of the monocyte and macrophage lineage. Ramprasad et al. (1995) [47] suggest the role of CD68 in lipid metabolism and phagocytosis. The CD68-positive cells expressed pro-inflammatory cytokines IL-1α, IL-6, and TNFα in the brain-damaged perinecrotic area [47,48,49]. CD68-positive cells play a key role in pro-inflammatory cytokine production in several diseases and are found in various tissues when they contribute to inflammation [50,51,52]. CD68-positive cells are an important source of inflammatory cytokines in several diseases.

All the factors mentioned above are linked with local and systemic inflammation; these characteristics impact local tissue healing, remodeling, and health. All of them were previously connected with other inflammatory diseases; therefore, it is crucial to understand the expression of these factors in maxillofacial pathologies such as OFC.

Research on inflammatory biomarker expression in bilateral cleft lip and palate remains underdeveloped. While cleft lip and palate as a general category is the most studied congenital craniofacial anomaly, the bilateral subtype specifically, which is the most severe and rarest form, affecting less than 10% of all cleft cases, remains absent from immunohistochemical research [53]. Furthermore, no studies have ever compared different dentition stages.

## 2. Materials and Methods

### 2.1. Material Characteristics of Subjects

This study was performed in accordance with the 1975 Helsinki Declaration, as well as independently reviewed and approved by the Ethical Committee of Rīga Stadiņs University (7 May 2003). All parents and patients were fully informed about the aim and nature of this study, and they provided written informed consent for participation in the study and its publication.

### 2.2. Selection Criteria of Patient Tissue Samples

Samples were included according to the following criteria: diagnosis of non-syndromic Cheilognathouranoschisis bilateralis, absence of clinically visible inflammation, absence of additional pathologies, no other congenital disease, and indications for bilateral cheiloplasty.

Samples were excluded according to the following criteria: signs of clinically visible active inflammation, proven congenital cleft palate, other congenital disease, and contraindications for bilateral cheiloplasty.

### 2.3. Characteristics of Patient Tissue Samples

In total, 14 patient samples, 12 of which were from males and 2 from females, were obtained during chelioplastic surgery in the Cleft Lip and Palate Center of the Institute of Stomatology of Rīga Stradiņš University. All the samples were from patients diagnosed with Cheilognathouranoschisis bilateralis aged 2 months to 12 years, thus including both milk and mixed dentition age, which allowed for two separate groups. The milk dentition age group included 9 children, with 6 male and 3 female patients, aged from 2 months to 1 year of age, whereas the mixed dentition age group included 5 male children aged 8 years to 12 years. All patients underwent either bilateral lip plastic, lip plastic, or bilateral cheiloplasty surgery.

### 2.4. Characteristics of Control Tissue Samples

In total, 10 control samples were obtained to form two distinct control groups with milk dentition age and mixed dentition age. All control group patients were fully informed about the duration, goals, and methods of the study and provided documentary agreement to the participation.

Milk dentition age group included 5 children aged 2 months to 2 years, whereas mixed dentition age group included 5 children aged 8 to 12 years. Milk dentition age group materials were gathered during upper frenula surgery, whereas mixed dentition age group tissue was gathered during tooth extraction surgery.

### 2.5. Hematoxylin and Eosin Staining

Routine staining of the samples was first performed in accordance with the hematoxylin and eosin staining technique used at the Institute of Anatomy and Anthropology of Riga Stradins University. First, tissue material was fixed for 24 h using 2% formaldehyde, 0.2% picric acid, and 0.1M phosphate buffer (pH 7.2). Second, tissue material was processed for 12 h using Tyrode’s buffer with 10% saccharose. Third, preservation of the tissue material in paraffin and cutting with a microtome into 5–7 μm thin samples were done. Lastly, samples were stained with hematoxylin and eosin [54].

### 2.6. Immunohistochemical Staining

Immunohistochemical staining of the patient samples from the patient and control groups was done utilizing standard streptavidin and biotin immunostaining for the identification of the cells and tissue containing GNLY, Resistin, FCGR1A, NF-kßp65 and CD68 molecules, as well as for the evaluation of the expression levels [54].

As with the hematoxylin and eosin staining, first, tissue material was fixed for 24 h using 2% formaldehyde, 0.2% picric acid, and 0.1M phosphate buffer (pH 7.2). Second, tissue material was processed for 12 h using Tyrode’s buffer with 10% saccharose. Third, preservation of the tissue material in paraffin and cutting with a microtome into 5-7 μm samples were done. Lastly, deparaffinization was performed and the biotin–streptavidin immunostaining method was implemented to visualize the presence of the previously mentioned proteins [54]. Table 1 summarizes the antibodies that were used.

Positive and negative controls were also done according to manufacturer guidelines and suggestions.

### 2.7. Evaluation of Factor Quantity

A semi-quantitative method was used to determine the relative expression of the mentioned proteins/factors. Evaluation of the protein expression in the visible field was performed based on the identifiers summarized in Table 2. The evaluation of the data was carried out independently by two morphologists. The evaluation was blind, and both morphologists later agreed on the median values, ensuring that the results were correct. For the evaluation, a Leica DC 300F digital camera was used (Leica Microsystems Digital Imaging, Cambridge, UK). Slide pictures were processed via the Image Pro Plus program (Media Cybernetics, Inc., Rockville, MD, USA).

### 2.8. Statistical Analysis

IBM SPSS (Statistical Package for the Social Sciences) software version 31.0 (IBM Company, Chicago, IL, USA) was used for statistical analyses of the data. The significance level was selected at a *p*-value < 0.05 and was used for all the tests and evaluations.

The semi-quantitative method produced new, original data that was non-numeric but arranged in a specific and unchangeable order; therefore, descriptive statistics, analytical statistics and non-parametric tests were used to evaluate correlation levels and statistical significance.

The Chi-square test was used for the detection of the statistically significant difference in the protein expression levels between the control and patient groups. If more than 20% of the cells had an expected count less than 5, then the Fisher–Freeman–Halton exact test was used for the precision of the *p*-value.

Spearman’s rank correlation was used for the detection of a possible statistically significant correlation between the expression of the two protein levels in the patient or control group. Statistical evaluation comparing mixed and milk dentition groups separately was also done using both mentioned tests.

The strength of the correlation between factors was interpreted using the following definition of Spearman’s rho (r_s_) values:

A very weak correlation: r_s_ = 0.00–0.19;

A weak correlation: r_s_ = 0.20–0.39;

A moderate correlation: r_s_ = 0.40–0.59;

A strong correlation: r_s_ = 0.60–0.79;

A very strong correlation: r_s_ = 0.80–1.00 [56].

Statistical data was also analyzed and independently approved by the staff of the Statistics Unit of the RSU Faculty of Medicine to ensure the correctness and credibility of the data.

## 3. Results

### 3.1. Routine Staining Morphological Evaluation of Control and Patient Samples

**Figure 1:**
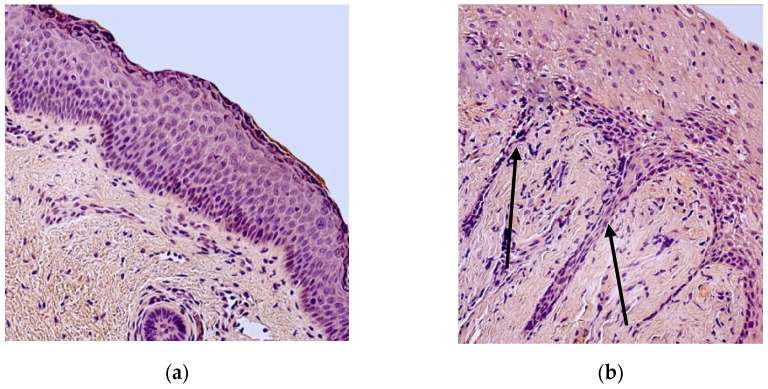
Hematoxylin and eosin staining of (**a**) control sample with morphologically normal oral cavity mucosa and no signs of severe inflammatory cell infiltration or other pathologies, and (**b**) mixed dentition stage patient sample—note the hyperplasia of the basal cells and imprint of the epithelium into connective tissue (arrows)—200×.

The samples of the control group revealed morphologically normal oral cavity mucosa with no significant signs of inflammatory cell infiltration or other pathologies (Figure 1).

The patient group presented significant morphological changes. Patient samples showed hyperplasia of the basal cells and severe inflammatory cell infiltration in epithelium and subepithelial tissues. A more pronounced epithelial invagination into the *lamina propria* was also noted (Figure 1).

### 3.2. Appearance and Distribution of Resistin

**Figure 2:**
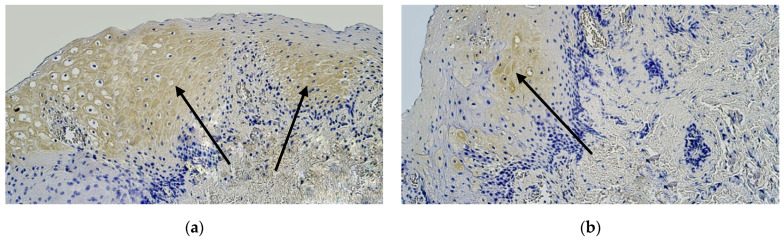
Immunohistochemistry of the Resistin-positive structures in the epithelium of milk dentition age patient and control samples. (**a**) Patient sample with moderate (+) number of Resistin-positive structures (arrow), 200×; (**b**) control sample with occasional (0/+) Resistin-positive structures (arrow), 200×.

In the milk dentition age patient group, the median number of Resistin-positive cells was occasional (0/+) in epithelium, and none (0) in connective tissues and salivary glands. In the milk dentition age control group, the median number of Resistin-positive cells was few (+) in epithelium, and none (0) in the connective tissue and salivary glands (Table 3). In the mixed dentition age patient group, the median number of Resistin-positive structures was none (0) in all the analyzed structures. In the mixed dentition age control group, the median number of Resistin-positive structures was few (+) in epithelium tissue and none (0) in connective tissue and salivary glands (Figure 2).

Comparison of the milk dentition age control group and milk dentition age patient group using the exact test revealed no statistical difference in epithelium, $\chi^{2}\left(N=14\right)=5.988,\;p=0.073$; connective tissue, $\chi^{2}\left(N=13\right)=2.134,\;p=0.478$; and salivary glands (expression of Resistin in both groups was equal to none (0)). Comparison of the mixed dentition age control group and mixed dentition age patient group also revealed no statistical difference in epithelium, $\chi^{2}\left(N=14\right)=4.452,\;p=0.167$; connective tissue, $\chi^{2}\left(N=13\right)=1.379,\;p\;>0.999$; and salivary glands (expression of Resistin in both groups was equal to none (0)).

### 3.3. Appearance and Distribution of GNLY

**Figure 3:**
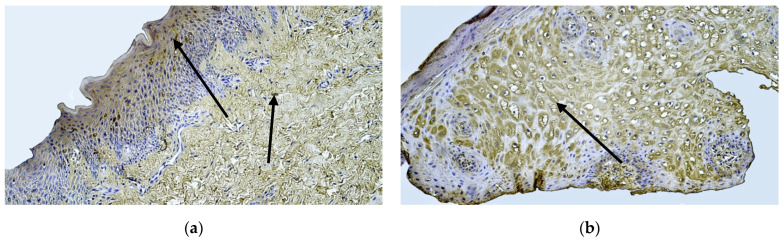
Immunohistochemistry of the GNLY-positive structures of mixed dentition age patient and control samples. (**a**) Patient sample with moderate (++) number of GNLY cells in epithelium and few (+) in connective tissue (arrows), 200×; (**b**) control sample with moderate (++) number of GNLY cells in epithelium (arrow), 200×.

In the milk dentition age patient group, the median number of GNLY-positive cells was few to moderate (+/++) in epithelium, few (+) in connective tissue, and none (0) in salivary glands. The milk dentition age control group presented a median number of positive structures of occasional (0/+) in epithelium, few (+) in connective tissue, and occasional (0/+) in salivary glands (Table 4). In the mixed dentition age patient group, the median number of GNLY-positive cells was few (+) in epithelium, as well as in the connective tissue and salivary glands. The mixed dentition age control group also showed consistent median expression equal to moderate (++) in epithelium tissue, connective tissue and salivary glands (Figure 3).

Comparing the milk dentition age patient and control groups using the Fisher–Freeman–Halton exact test, no statistically significant correlation was detected in epithelium, $\chi^{2}\left(N=14\right)=7.350,\;p=0.301$; connective tissue, $\chi^{2}\left(N=13\right)=3.943,\;p=0.249$; and salivary glands, $\chi^{2}\left(N=10\right)=5.085,\;p=0.133$. Comparison of the mixed dentition age group revealed no statistically significant correlation in epithelium, $\chi^{2}\left(N=14\right)=2.996,\;p=0.810$; connective tissue, $\chi^{2}\left(N=13\right)=6.462,\;p=0.079$; and salivary glands, $\chi^{2}\left(N=10\right)=4.294,\;p=0.100$.

### 3.4. Appearance and Distribution of FCGR1A

**Figure 4:**
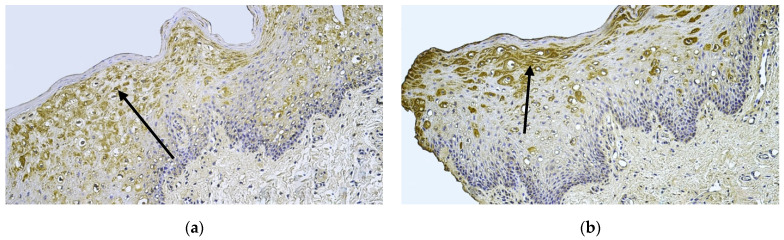
Immunohistochemistry of the FCGR1A-positive structures of milk dentition age patient and control samples. (**a**) Patient sample with moderate (++) expression in the epithelial tissue (arrow), 200×; (**b**) control sample with numerous (+++) expression in the epithelial tissue (arrow), 200×.

In the milk dentition age patient group, the median number of FCGR1A-positive cells was moderate (++) in epithelium and salivary glands; meanwhile, the median number of positive cells was few (+) in connective tissue. The milk dentition age control group also presented a median of a moderate (++) number of cells in epithelium and salivary glands. The median number of FCGR1A-positive cells in connective tissue was few to moderate (+/++) (Table 5). In the mixed dentition age patient group, the median number of FCGR1A-positive cells was moderate (++) in epithelium and salivary glands; meanwhile, the median number of FCGR1A-positive cells in connective tissue was few (+). The median number of the FCGR1A-positive cells in the control group was moderate (++) in epithelium, few to moderate (+/++) in connective tissue, and few (+) in salivary glands (Figure 4).

The milk dentition age patient and control group comparison revealed no statistically significant correlations in epithelium, $\chi^{2}\left(N=14\right)=4.298,\;p=0.215$; connective tissue, $\chi^{2}\left(N=13\right)=4.474,\;p=0.467$; and salivary glands, $\chi^{2}\left(N=10\right)=2.570,\;p=0.750$. A comparison of the mixed dentition age patient and control groups revealed no statistically significant correlation in the epithelium, $\chi^{2}\left(N=14\right)=2.884,\;p>0.999$, or salivary glands, $\chi^{2}\left(N=10\right)=2.903,\;p>0.999$. Additionally, in connective tissue, a statistically significant correlation was detected, $\chi^{2}\left(N=13\right)=7.606,\;p=0.016,$ and indicated that the FCGR1A level in the patient group was decreased.

### 3.5. Appearance and Distribution of CD68

**Figure 5:**
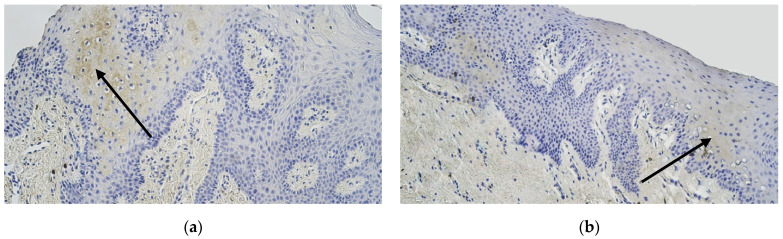
Immunohistochemistry of the CD68-positive structures of the milk dentition age group patient sample; (**a**) and (**b**) show few (+) positive expressions in the epithelial tissue (arrows), 200×.

In the milk dentition age patient group, the median number of CD68-positive structures was none (0) in connective tissue and salivary glands; however, the median number of CD68-positive cells was few (+) in epithelium. The milk dentition age control group presented a median number of CD68-positive structures equal to none (0) in the epithelium and salivary glands. The median number of CD68-positive cells in connective tissue was few (+) (Table 6). In the mixed dentition age patient group, the median number of CD68-positive structures was few (+) in the epithelial tissue, occasional (0/+) in connective tissue, and none (0) in salivary glands. The median number of CD68-positive cells in the control group was occasional (0/+) in epithelium, and none (0) in connective tissue and salivary glands (Figure 5).

A comparison of patient samples and controls within the milk dentition age group revealed no statistically significant correlations in epithelium, $\chi^{2}\left(N=14\right)=1.842,\;p=0.728$; connective tissue, $\chi^{2}\left(N=13\right)=3.508,\;p=0.172$; and salivary glands (no expression of CD68 was detected in either group). Comparison of the mixed dentition age patient and control group revealed no statistically significant correlation in connective tissue, $\chi^{2}\left(N=13\right)=0.400,\;p>0.999,$ or salivary glands (no expression of CD68 was detected in either group). However, in the epithelial tissue, a statistically significant correlation was detected, $\chi^{2}\left(N=14\right)=9.057,\;p=0.048,$ and indicated that the CD68 level in the patient group tends to be increased.

### 3.6. Appearance and Distribution of NF-kβ p65

In the milk dentition age patient group, the median number of NF-kβp65-positive cells was occasional (0/+) in connective tissue and salivary glands; however, the median number of NF-kβp65-positive cells was few to moderate (+/++) in epithelium. The milk dentition age control group presented a median number of few (+) positive cells in epithelium and connective tissue. The median number of NF-kβp65-positive cells in salivary glands was moderate (++) (Table 7).

**Figure 6:**
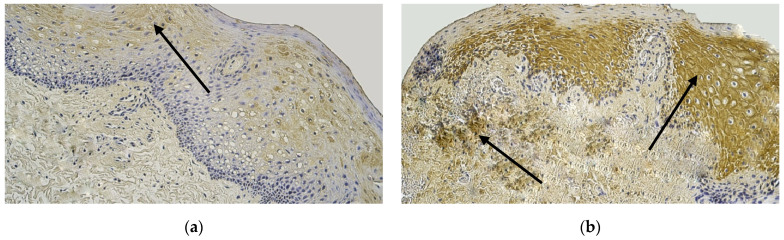
Immunohistochemistry of the NF-kβp65-positive structures of the mixed dentition age patient and control samples; (**a**) shows the patient sample with few (+) expressions of NF-kBp65 in the epithelial tissue (arrows), 200×; (**b**) shows moderate (++) positive expression in the epithelial tissue and few (+) expressions in the connective tissue (arrows), 200×.

In the mixed dentition age patient group, the median of NF-kβp65-positive cells was few (+) in connective tissue and salivary glands, but few to moderate (+/++) in epithelium. The median number of NF-kβp65-positive cells in the control group was moderate (++) in epithelium, few (+) in connective tissue, and none (0) in salivary glands (Figure 6).

A comparison of the patients of milk dentition age and the control group revealed no statistically significant correlations in epithelium, $\chi^{2}\left(N=14\right)=6.500,\;p=0.221$. However, in connective tissue, a statistically significant correlation was detected, $\chi^{2}\left(N=13\right)=6.971,\;p=0.043,$ and indicated that the NFkβp65 level in the patient group tends to be a little decreased. A statistically significant difference in the appearance of NFkBp65 in the salivary glands was detected, $\chi^{2}\left(N=10\right)=7.462,\;p=0.017,$ and indicated that the NFkβp65 level in the patient group tends to be a little decreased as well. A comparison of the mixed dentition age patient and control groups revealed no statistically significant correlation in epithelium, $\chi^{2}\left(N=14\right)=6.180,\;p=0.143$; connective tissue, $\chi^{2}\left(N=13\right)=1.111,\;p>0.999;$ and salivary glands, $\chi^{2}\left(N=10\right)=5.000,\;p=0.100$.

**Table 7:** Expression intensity of NF-kBp65 in all analyzed samples with median values and *p*-values for (**a**) the milk dentition age group and (**b**) the mixed dentition age group.

(a) Milk Dentition Age				(b) Mixed Dentition Age			
Sample Number	ET	CT	SG	Sample Number	ET	CT	SG
Patient Median	+/++	0/+	0/+	Patient Median	+/++	+	+
Control Median	+	+	++	Control Median	++	+	0
*p*-Value	0.221	0.043 *	0.017 *	*p*-Value	0.143	>0.999	0.100
$\chi^{2}$ Value	6.500	6.971	7.462	$\chi^{2}$ Value	6.180	1.111	5.000

Abbreviations: ET—Epithelial tissue; CT—connective tissue; SG—salivary glands. * *p* < 0.05.

### 3.7. Comparison of Inflammatory Proteins/Factors in Patient and Control Tissue

**Figure 7:**
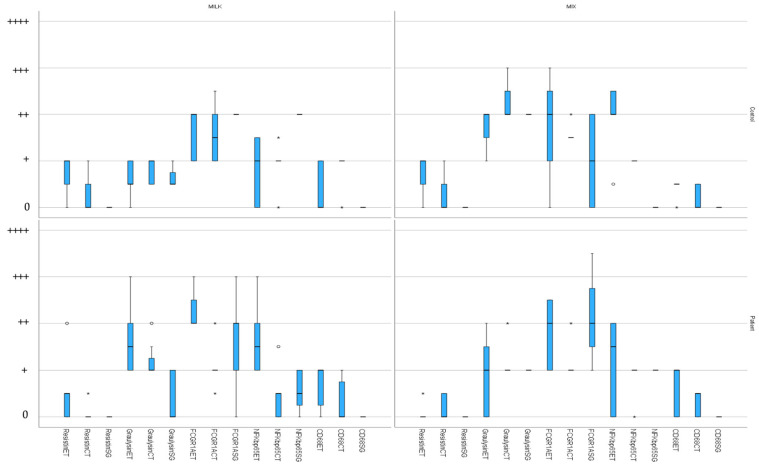
Median expression intensity of all analyzed factors in all analyzed tissues in the milk dentition age group, comparing the patient and control groups. Abbreviations: ET—epithelial tissue; CT—connective tissue; SG—salivary glands; Starred point—extreme outliner; Circle point—outliner.

Figure 7 illustrates a visual summary of the median expression intensity of all analyzed inflammatory factors and proteins, comparing the patient and control groups in all analyzed tissue samples.

### 3.8. Correlation Between Inflammatory Proteins/Factors in Patient-Group-Analyzed Structures

Spearman’s correlation analysis demonstrated eight very strong relationships among the examined factors (r_s_ = 0.80–1.00) and one strong relationship among the examined factors (r_s_ = 0.60–0.79). Table 8 presents the very strong/strong positive correlations between inflammatory proteins/factors. Two correlations were detected in the milk dentition age group and seven in the mixed dentition age group.

GNLY and FCGR1A both showed the highest number (five) of significant Spearman’s correlations. CD68 showed four statistically significant correlations and NF-kβp65 showed three statistically significant correlations. Resistin showed the least number of correlations, at only one.

## 4. Discussion

This study analyzes the expression of Resistin, Granulysin, FCGR1A, CD68, and NF-kβp65 comparing the number of positive structures/cells between control and patient groups in two separate dentition stages. In the milk dentition age group NF-kβp65 was shown to be statistically significantly decreased in the connective tissue and salivary glands of the patient group. However, in the mixed dentition age group FCGR1A was decreased in the patient group connective tissue, while CD68 was increased in the patient group epithelium tissue. Other studied factors did not show a statistically significant difference between patient and control groups; however, some of them showed a strong association with other studied proteins/factors.

NF-kβ is a widely known transcription factor that induces many cell regulatory processes, inducing inflammation, fibrosis, and tissue remodeling [35]. The expression of the NF-kβp65 subunit can be induced by many different factors, including cytokines [36], hormones [37], chemical compounds [38], cellular stress [39] and others. NF-kβ functions as a homo- or heterodimer, which can be formed of different subunits [41]. In our study we analyze only one structural unit, p65, which makes the RelA domain of the NF-kβ structure. The p65 subunit has been proven to play a key role in the regulation of many cellular processes; for instance, p65, when binding to the p50 NF-kβ domain, promotes expression of several pro-inflammatory cytokines such as TNFα, IL-1β and IL-6 [35]. Thus, it severely increases local inflammation in the impacted area and areas of increased p65:p50 dimer expression. Nevertheless, the p65 subunit by itself can promote the expression of IL-12, which some studies have suggested plays a pro-inflammatory role [45]. Yet some other scientific works emphasize the effect of the p65 subunit as being anti-inflammatory, and in some mouse studies, p65 has shown increased induction of anti-inflammatory mediators and increased expression of anti-inflammatory genes. The p65 subunit’s connection with aryl hydrocarbon receptors (AhRs) leads to ubiquitination and proteasomal degradation of pro-inflammatory cytokines, thus actually suppressing the immune response inside the impacted area [45]. Bidirectional functions of the studied subunit can also indicate bidirectional response; it is worth considering both when talking about the inflammation levels in the clefted tissues. On the one hand, decreased expression in the milk dentition age group inside connective tissue and salivary glands can indicate a weakened immune response in the mentioned areas via the NF-kβ transcriptional factor and suggest a potentially different inflammatory base in impacted areas. On the other hand, the p65 subunit could potentially act as anti-inflammatory; thus, a decrease in appearance can indicate much larger expression of pro-inflammatory cytokines because their degradation is also hugely decreased.

The mixed dentition age group showed decreased expression of FCGR1A in the connective tissue of the patient group. The FCGR1A gene encodes CD64, which is a widely studied high-affinity IgG Fc receptor on the surface of monocytes, macrophages, and dendritic cells [31]. CD64 plays a key role in inflammation via stimulation of pro-inflammatory cytokine production and NF-kβ transcription pathway activation [57]. After antigen binding to CD64, phagocytosis and degranulation are induced, and involvement of CD64 with the target molecule activates intracellular signaling pathways, such as the NF-kβ pathway, which also leads to the production of pro-inflammatory cytokines [57]. Several inflammatory conditions have been proven to be associated with increased expression of the CD64 molecule, such as sarcoidosis, proliferating diabetic retinopathy, and rheumatoid arthritis [31,34,58]. The role of CD64 in inflammation is unambiguous, and the decreased expression in the connective tissue that is suggested by our data could possibly mean that in the older cleft-palate patient group, the number of activated monocytes and macrophages within the connective tissue is reduced. This finding may indicate that in the connective tissue, a weakened immune response could compromise the organism’s capacity for pathogen clearance and immune complex handling.

CD68 expression in the mixed dentition age group epithelium was found to be statistically significantly increased in patient samples compared to controls. CD68 is a member of the lysosome-associated membrane protein (LAMP) family that is restricted in its expression to cells of the monocyte and macrophage lineage. CD68-positive cells play a key role in pro-inflammatory cytokine production. CD68-positive cells are an important source of inflammatory cytokines in several diseases [52]. CD68-positive cells express pro-inflammatory cytokines IL-1α, IL-6 and TNFα in different human body regions [49]. Other studies describe that CD68-positive macrophages increased significantly in Parkinson’s disease. The increased amount contributed to inflammation and increased macrophage infiltration [51]. Our data suggest that the CD68 level in epithelial tissue in patient samples is increased. Based on the above-mentioned facts, a potential increase in epithelial expression can indicate intensification of inflammation in the clefted epithelial-border tissue.

It is interesting to note that FCGR1A and CD68 are both mainly associated with macrophages and monocyte expression. FCGR1A, however, shows decreased expression in the connective tissue, while CD68 in epithelial tissue is increased. This association can potentially indicate some decompensation of the monocyte–macrophage system in older cleft-affected connective tissue, with some stimulation of defense cells from the side of the epithelium.

When analyzing the interaction between inflammatory proteins/factors, two statistically significant correlations were detected in the milk dentition age group, one of them being very strong and one being strong. However, the number of detected interactions in the mixed dentition age group markedly increased to seven. This finding potentially indicates the intensification of the signaling pathways and inflammation character with age. GNLY and FCGR1A both showed the biggest number of interactions. GNLY protein is a member of the saposin-like protein family. Its structure suggests a potential mechanism of action by which GNLY functions as a lytic molecule [13,14,15,16,17]. GNLY levels in systemic inflammation disease have been proven to be increased [21,22,23]. However, data on GNLY expression levels in cleft-lip and -palate tissue is currently lacking. Based on the above-mentioned facts, an increased number of interactions with other proteins/factors suggests a potential GNLY role in regulating the expression of other inflammatory factors. Yet it is hard to make a conclusion about the increased microbial presence in clefted tissues, as the number of positive cells between patients and controls did not differ significantly. FCGR1A has been proven to be decreased in the connective tissue of the patient group; however, based on Spearman’s correlation rank in the patients with preserved expression of FCGR1A, its interaction with other protein/factors is very high, suggesting its potential role in inflammatory regulation.

Our data suggest a potential increase in inflammation in clefted tissues. Increased expression of CD68 in epithelial cells in the mixed dentition age group could potentially indicate increased macrophage infiltration of the epithelial barrier, a classic sign of chronic inflammation in tissues. Furthermore, the strong positive correlations between GNLY, FCGR1A, NF-kβp65 and CD68 in epithelial cells confirm a coordinated activation of the inflammatory network, where cytotoxic lymphocyte-mediated defense, macrophage recruitment and NF-kβ pro-inflammatory signaling act simultaneously to create a persistent inflammatory microenvironment in the cleft-affected tissues.

Further studies are needed to understand not only the NF-kβp65 inflammatory role through the analysis of other NF-kβ subunit expressions in both the milk and mixed dentition age groups. Furthermore, additional studies on CD68 and FCGR1A are needed, as both are markers of monocytes and macrophages. Interestingly, our study showed that one is increased (CD68 in ET) while the other is decreased (FCGR1A in CT).

Our study had several important limitations that may have influenced the results and limited the interpretation of the observed correlations.

The most important limitation of our study is the small number of patient and control samples. The bilateral cleft-lip and -palate cases analyzed in this study are the rarest congenital cleft-lip and -palate variation, which is why even analysis of a small number of patient samples is very valuable. The limitation in control tissue samples is associated with ethical concerns, as access to relatively healthy tissue from unaffected cleft tissues is very limited due to the lack of surgical concerns regarding the retrieval of any relatively healthy oral cavity tissue. It is important to note that other confounding factors can affect the results, as control samples were collected during clinical procedures, and the underlying indication for those procedures may still influence local tissue biology. Furthermore, factors such as oral hygiene, medication use and nutritional status can influence the results as well. An additional limitation of our study is an imbalance in the analyzed groups, such as gender inequality, as males are represented in much larger numbers, which may influence the data. Between genders, inflammatory or immune responses can vary. It is important to mention that cleft-lip and -palate is much more prevalent in boys than in girls, and sexual inequality has been proven in many studies [53,59].

The semi-quantitative method of sample evaluation can be subjective; however, for the best results, two independent morphologists evaluated the tissue samples. Evaluation was completely blind. Later, both agreed on the median values, ensuring that the results were correct. Future research might benefit from detecting factor concentrations using ELISA and specific gene analysis (by in situ hybridization), thereby ensuring the objective evaluation of the appearance of the studied proteins.

Furthermore, our study is cross-sectional, meaning only associations can be observed from the collected data; additional research is crucial to determine causal relationships among inflammatory markers.

## 5. Conclusions

The reduced expression of NF-kβp65 in the connective tissue and salivary glands of the milk dentition group could possibly indicate that the inflammatory activity may vary via different mechanisms depending on the dentition stage.

The decrease in FCGR1A expression in the connective tissue of the mixed dentition patients and the increase in CD68 expression in the epithelium of the same group can be interpreted as decompensation of the monocyte–macrophage system in older cleft-affected connective tissue, with some stimulation of defense cells from the side of the epithelium.

The appearance of a significantly higher number of Spearman’s very strong and strong correlations between inflammatory factors in the cleft-affected mixed dentition age group of children suggests the intensification of signal pathways and probably of inflammation character with age. GNLY and FCGR1A showed the highest number of significant Spearman’s correlation ranks, indicating their strong association with other inflammatory proteins.

## Figures and Tables

**Table 1:** The serial number, dilution, developer company and country of all the used antibodies.

Type of Protein	Serial Number	Dilution	Company	Location
Resistin	Orb1151827	1:100	Biorbyt LTD	Cambridge, UK
GNLY	Orb1417601	1:100	Biorbyt LTD	Cambridge, UK
FCGR1A	Orb228465_4	1:200	Biorbyt LTD	Cambridge, UK
CD68	Cmc16829040	1:100	Cell Marque	California, USA
NF-kβ p65	Orb37069	1:100	Biorbyt LTD	Cambridge, UK

**Table 2:** The identifiers used in the study and their explanation [55,56].

Identifier	Explanation
0	No positive structures (0%)
0/+	Occasional positive structures (12.5%)
+	Few positive structures (25%)
+/++	Low-to-moderate number of positive structures (37.5%)
++	Moderate number of positive structures (50%)
++/+++	Moderate-to-high number of positive structures (62.5%)
+++	Numerous positive structures (75%)
+++/++++	Numerous-to-abundant positive structures (87.5%)
++++	Abundance of positive structures (100%)

**Table 3:** Expression intensity of the Resistin median values and *p*-values for (**a**) in the milk dentition age group and (**b**) mixed dentition age group.

(a) Milk Dentition Age				(b) Mixed Dentition Age			
Sample Number	ET	CT	SG	Sample Number	ET	CT	SG
Patient Median	0/+	0	0	Patient Median	0	0	0
Control Median	+	0	0	Control Median	+	0	0
*p*-Value	0.073	0.478	-	*p*-Value	0.167	>0.999	-
$\chi^{2}$ Value	5.988	2.134	-	$\chi^{2}$ Value	4.452	1.379	-

Abbreviations: ET—Epithelial tissue; CT—connective tissue; SG—salivary glands.

**Table 4:** Expression intensity of GNLY in all analyzed samples with median values and *p*-values for (**a**) the milk dentition age group and (**b**) the mixed dentition age group.

(a) Milk Dentition Age				(b) Mixed Dentition Age			
Sample Number	ET	CT	SG	Sample Number	ET	CT	SG
Patient Median	+/++	+	0	Patient Median	+	+	+
Control Median	0/+	+	0/+	Control Median	++	++	++
*p*-Value	0.301	0.249	0.133	*p*-Value	0.810	0.079	0.100
$\chi^{2}$ Value	7.350	3.943	5.085	$\chi^{2}$ Value	2.996	6.462	4.294

Abbreviations: ET—Epithelial tissue; CT—connective tissue; SG—salivary glands.

**Table 5:** Expression intensity of FCGR1A in all analyzed samples with median values and *p*-values for (**a**) the milk dentition age group and (**b**) the mixed dentition age group.

(a) Milk Dentition Age				(b) Mixed Dentition Age			
Sample Number	ET	CT	SG	Sample Number	ET	CT	SG
Patient Median	++	+	++	Patient Median	++	+	++
Control Median	++	+/++	++	Control Median	++	+/++	+
*p*-Value	0.215	0.467	0.750	*p*-Value	>0.999	0.016 *	>0.999
$\chi^{2}$ Value	4.298	4.474	2.570	$\chi^{2}$ Value	2.884	7.606	2.903

Abbreviations: ET—Epithelial tissue; CT—connective tissue; SG—salivary glands. * *p* < 0.05.

**Table 6:** Expression intensity of CD68 in all analyzed samples with median values and *p*-values for (**a**) the milk dentition age group and (**b**) the mixed dentition age group.

(a) Milk Dentition Age				(b) Mixed Dentition Age			
Sample Number	ET	CT	SG	Sample Number	ET	CT	SG
Patient Median	+	0	0	Patient Median	+	0/+	0
Control Median	0	+	0	Control Median	0/+	0	0
*p*-Value	0.728	0.172	-	*p*-Value	0.048 *	>0.999	-
$\chi^{2}$ Value	1.842	3.508	-	$\chi^{2}$ Value	9.057	0.400	-

Abbreviations: ET—Epithelial tissue; CT—connective tissue; SG—salivary glands. * *p* < 0.05.

**Table 8:** Spearman’s correlation ranks and *p*-values between analyzed proteins and factors.

Dentition Group	Correlation Between Factors	r_s_	*p*-Value
Milk	Resistin ET ↔ CD68 ET	0.904	0.002
	GNLY SG ↔ FCGR1A SG	0.756	0.049
Mixed	GNLY ET ↔ FCGR1A ET	0.973	0.005
	GNLY ET ↔ FCGR1A SG	0.998	0.001
	GNLY ET ↔ NF-kβ p65 ET	0.973	0.005
	GNLY ET ↔ CD68 ET	0.889	0.044
	FCGR1A ET ↔ NF-kβ p65 ET	0.917	0.029
	FCGR1A ET ↔ CD68 ET	0.913	0.030
	NF-kβ p65 ET ↔ CD68 ET	0.914	0.030

Abbreviations: ET—Epithelial tissue; CT—connective tissue; SG—salivary glands.

## Data Availability

The original contributions presented in this study are included in the article. Further inquiries can be directed to the corresponding author.

## Abbreviations

The following abbreviations are used in this manuscript:

OFC	Orofacial clefts
GNLY	Granulysin
ET	Epithelial tissue
CT	Connective tissue
SG	Salivary glands
